# Portal vein thrombosis on unenhanced MRI: a case series

**DOI:** 10.1259/bjrcr.20220059

**Published:** 2022-10-27

**Authors:** Chau Hung Lee

**Affiliations:** 1 Department of Radiology, Tan Tock Seng Hospital, Singapore, Singapore

## Abstract

Portal vein thrombosis (PVT) is usually diagnosed on contrast-enhanced CT, MRI or Doppler ultrasound. However, for patients with contraindications to intravenous contrast, its diagnosis is challenging. In these patients, PVT can be detected on unenhanced MRI using T2, T1 and diffusion-weighted imaging. These sequences may also help differentiate between bland PVT, portal pyemia and tumour thrombus. This case series aims to highlight the various appearances of PVT on unenhanced MRI.

## Introduction

Portal vein thrombosis (PVT) is a condition where hepatopetal flow through the portal venous system is obstructed as a result of intraluminal obstruction, and can be complete or partial.^1^ Diagnosis of PVT can be made with contrast-enhanced CT and MRI or Doppler ultrasound. CT and MRI are usually preferred over Doppler ultrasound as they are less operator dependent, provide superior visualisation of vascular anatomy and adjacent structures, and can demonstrate aetiology of PVT.^2,3^ However, many patients may have concomitant renal impairment when contrast administration is contraindicated due to risks of contrast nephropathy (for iodinated contrast) or nephrogenic systemic fibrosis (for gadolinium-based contrast).

For such patients, unenhanced CT is virtually ineffective in diagnosing PVT. Doppler ultrasound can be utilised if there is clinical suspicion of PVT in patients who are unable to undergo contrast-enhanced CT or MRI. However, Doppler ultrasound suffers from variable image quality due to patient factors and operator-dependence, challenges from masses or prior intervention that can alter normal anatomy, as well as poor visualisation of splanchnic vessels.^4^ Studies have shown that PVT can be detected on unenhanced MRI (NC-MRI), and detection is improved with dedicated techniques such as T2*, black blood imaging, time-of-flight and time-spatial labelling inversion pulse techniques.^5,6^ However, as PVT is often an incidental finding, it is therefore important that radiologists be familiar with the various appearances of PVT on standard NC-MRI sequences.

**Figure 1.**
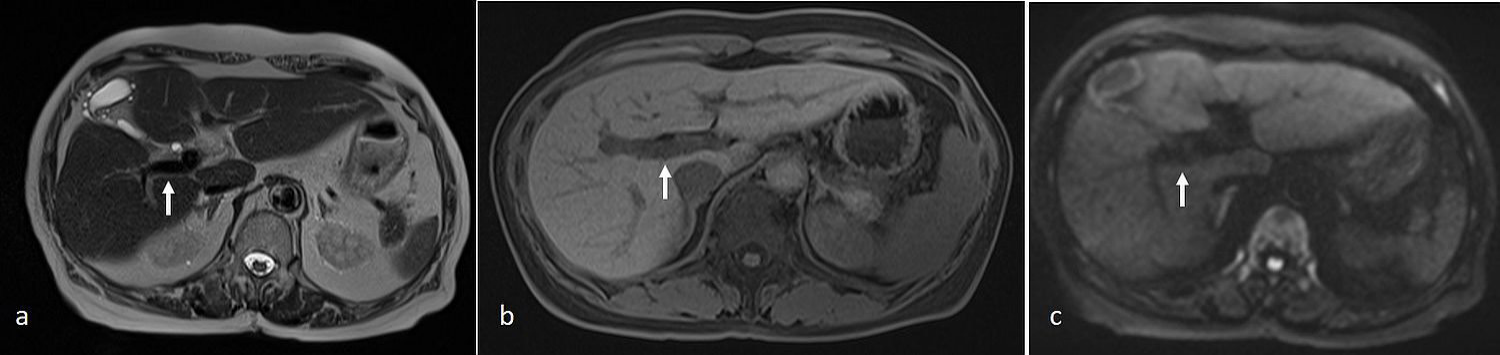
A 40-year-old gentleman underwent liver MRI for evaluation of raised serum alphafetoprotein on health screening. (**a**) Axial T2-SSFSE sequence showed normal flow-related signal void in the patent portal veins (arrow). (**b**) On T1 3D-VIBE, the portal veins appeared hypointense (arrow). (**c**) On high b-value DWI (*b* = 1000), the portal veins showed signal void (arrow). Portal veins were confirmed to be patent after intravenous administration of Dotarem (not shown). DWI, diffusion-weighted imaging; SSFSE, single-shot fast spin echo; VIBE, volume interpolated breath-hold examination.

The portal vein is formed by the confluence of the superior mesenteric and splenic veins posterior to the pancreatic neck. At the porta hepatis, it bifurcates into the right and left portal vein. The right portal vein bifurcates into the anterior and posterior branches while the left portal vein bifurcates into the transverse and umbilical branches.^7^ On *T*_2_ weighted imaging (*T*_2_WI), the patent portal vein demonstrates normal flow-related signal loss (“flow void”) due to fast flowing blood, appearing hypointense (Figure 1a). On *T*_1_ weighted imaging (*T*_1_WI), the patent portal vein appears hypointense to the adjacent liver parenchyma (Figure 1b). On diffusion-weighted imaging (DWI), signal from patent vessels is progressively suppressed with increasing diffusion gradient (b-values). On high b-value DWI, the patent portal vein demonstrates absent signal (Figure 1c).

In our institution (Tan Tock Seng Hospital, Singapore), abdominal MRI is performed on either a 3 T scanner (Magnetom Vida, Siemens Healthineers, Erlangen, Germany) or a 1.5 T scanner (Magnetom Sola, Siemens Healthineers, Erlangen, Germany; Ingenia, Phillips Healthcare, Netherlands; Signa HDxt, GE Healthcare, Milwaukee, WI, USA). Standard abdominal NC-MRI sequences include: *T*_2_ weighted single-shot fast spin echo (*T*_2_-SSFSE) in the axial and coronal planes, *T*_2_ weighted fast spin echo with fat-saturation in the axial plane either with breath-hold or respiratory-triggered (*T*_2_-FS), DWI with three b-values (0, 500, 1000 mm/s^2^), unenhanced *T*_1_ weighted 3D volume interpolated breath-hold examination with fat-saturation (*T*_1_ 3D-VIBE) in the axial plane. MRI parameters vary slightly between scanners and are briefly summarised in Table 1.

**Table 1.** Brief summary of NC-MRI parameters in our institution (Tan Tock Seng Hospital, Singapore)

Parameter	*T*_2_-SSFSE / *T*_2_-FS	DWI	*T*_1_ 3D-VIBE
TE (ms)	60–90	60–90	2–6
TR (ms)	1100–3300	1400–1800	4–8
Slice thickness (mm)	4–5	6	3–4
Flip angle (degrees)	90–180	90–180	10–20
In-plane resolution (mm)	0.6–1.2	1.3–1.5	0.5–0.7
FOV (mm)	34–38	34–38	34–38
Acceleration factor	2	2	2

DWI, diffusion-weighted imaging; FOV, field of view; SSFSE, single-shot fast spin echo; TE, echo time; TR, repetition time; VIBE, volume interpolated breath-hold examination.

This short case series aims to highlight features of PVT on NC-MRI, particularly *T*_2_WI, *TT*_1_WI and DWI.

## Case 1

**Figure 2.**
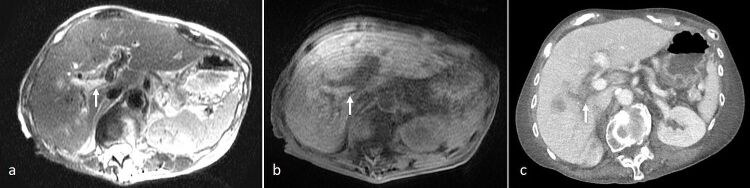
An 83-year-old lady with a history of rectal cancer, underwent radiofrequency ablation of a right hepatic lobe metastasis. She subsequently presented with fever and mild abdominal pain. NC-MRI was performed. (**a**) Axial *T*_2_-SSFSE showed “loss of signal void” in the right portal vein (arrow) compared to the left, appearing hyperintense. (**b**) On *T*_1_ 3D-VIBE, there was corresponding T1-hyperintensity (arrow). (**c**) Subsequent contrast-enhanced CT scan confirmed acute right PVT (arrow). PVT, portal vein thrombosis; SSFSE, single-shot fast spin echo; VIBE, volume interpolated breath-hold examination.

Patient A was an 83-year-old lady with a history of rectal cancer, who had undergone radiofrequency ablation of a right hepatic lobe metastasis. 1 week after the procedure, she presented with low-grade fever and mild right hypochondrial pain. Laboratory tests revealed leucocytosis with white cell count of 20.5 × 10^9^/L (4–9.6), raised alkaline phosphatase of 149 U L^−1^ (40–120) with normal serum bilirubin of 12 umol L^−1^. An MRI was performed to evaluate for possible liver abscess, in view of recent intervention. No intravenous contrast was administered due to severe renal impairment with eGFR of 23 mL/min/1.73 m^2^. NC-MRI showed loss of signal void on *T*_2_WI and hyperintensity on *T*_1_WI (Figure 2a and b). There was no significant restricted diffusion (not shown). Imaging diagnosis was that of acute PVT. Patient was hydrated with recovery of renal function over 4 days, to baseline eGFR of 45 mL/min/1.73 m^2^. Subsequent contrast-enhanced CT scan after recovery of renal function confirmed thrombosis of the right portal vein (Figure 2c).

## Case 2

**Figure 3.**
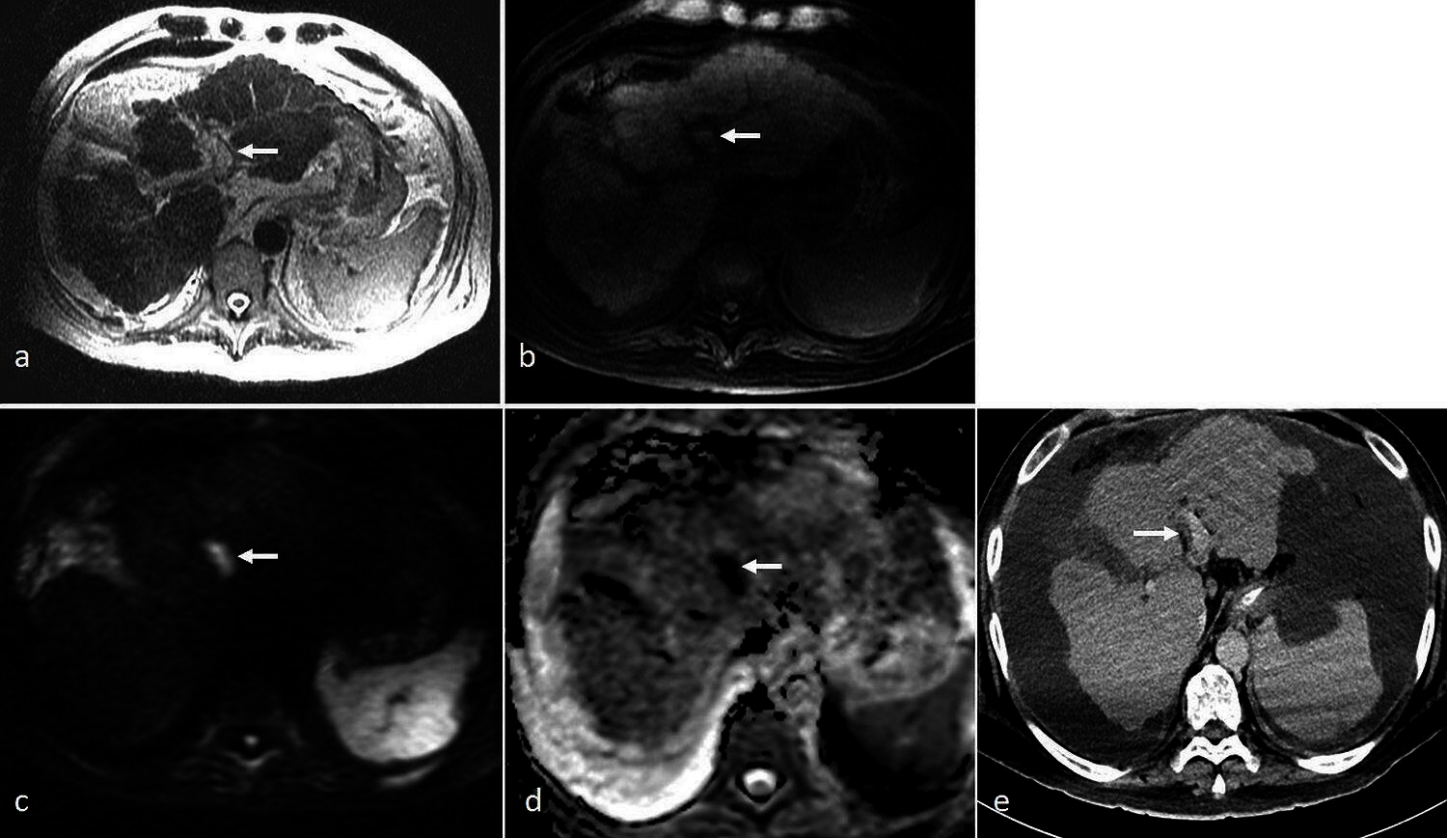
A 65-year-old lady with a history of advanced liver cirrhosis was admitted for acute cholecystitis and underwent a percutaneous cholecystostomy. Liver function tests showed hyperbilirubinemia, and MRCP was performed. (**a**) Axial *T*_2_-SSFSE showed “loss of signal void” in the left portal vein, appearing hyperintense. (**b**) On *T*_1_ 3D-VIBE, there was corresponding T1-hyperintensity (arrow). (**c**) On DWI (*b* = 1000), there was marked hyperintensity (arrow). (**d**) ADC map showed corresponding marked hypointensity (arrow), indicating restricted diffusion. (**e**) Contrast-enhanced CT scan performed 1 week earlier showed the left portal vein to be patent (arrow). This confirmed the acute left PVT on current MRI. ADC, apparent diffusion coefficient; DWI, diffusion-weighted imaging; PVT, portal vein thrombosis; SSFSE, single-shot fast spin echo; VIBE, volume interpolated breath-hold examination.

Patient B was a 65-year-old lady with a history of advanced liver cirrhosis secondary to chronic hepatitis. She was admitted for acute cholecystitis and underwent a percutaneous cholecystostomy. She was recovering uneventfully. However, about 1 week after the procedure, routine blood tests found a raised serum bilirubin of 72 umol L^−1^ (5–30). a magnetic resonance cholangiopancreaticography (MRCP) was performed to evaluate for biliary dilatation. At our institution (Tan Tock Seng Hospital, Singapore), this is performed without intravenous contrast. MRCP did not show significant biliary dilatation. However, along the left portal vein, there was loss of signal void on *T*_2_WI, hyperintensity on *T*_1_WI, as well as associated restricted diffusion (Figure 3a–d). Imaging diagnosis was that of acute PVT. Review of contrast-enhanced CT scan 1 week earlier at presentation, did not show significant thrombosis (Figure 3e), confirming acute nature of the PVT.

## Case 3

**Figure 4.**
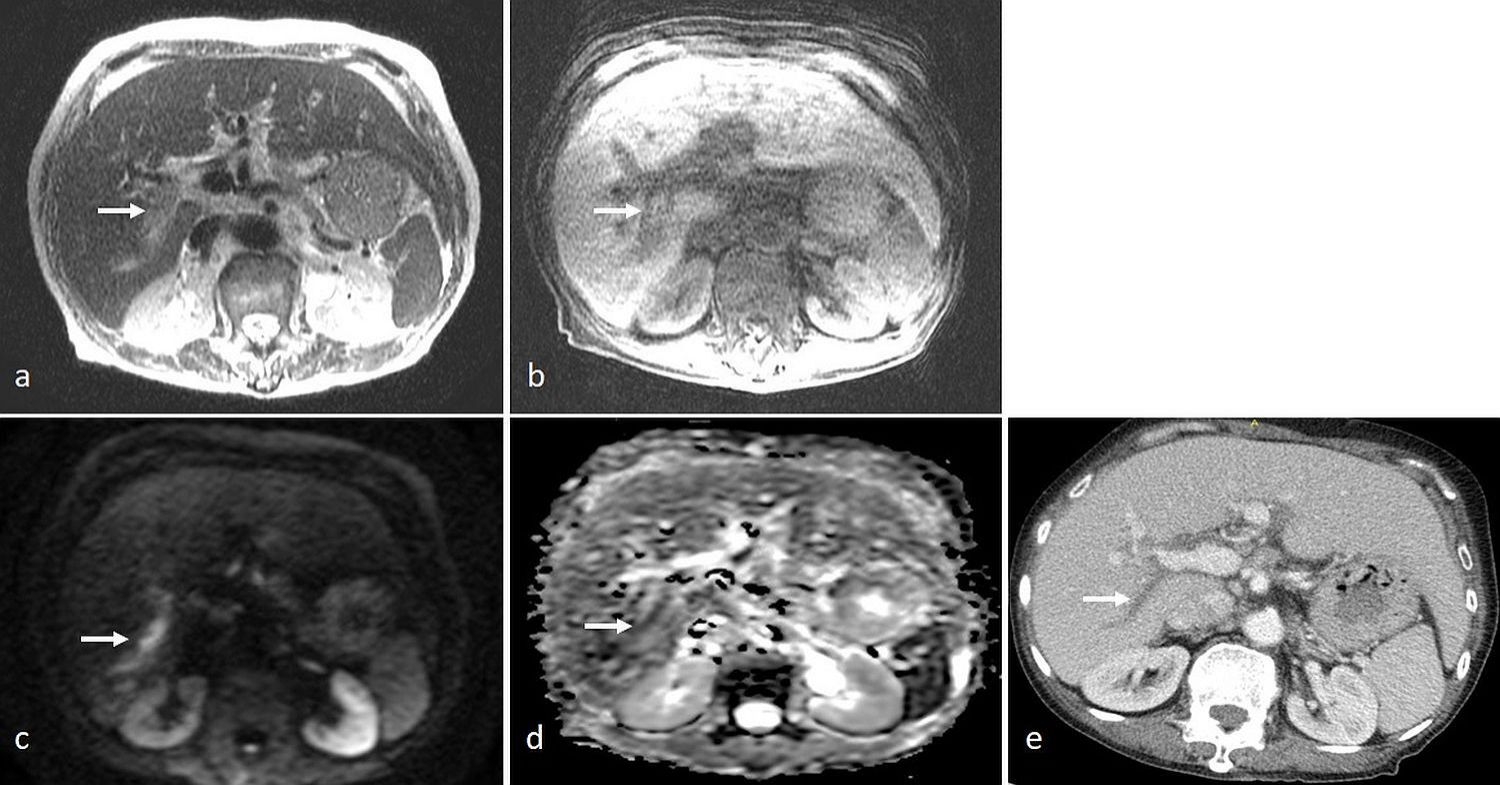
A 77-year-old lady presented with *E. coli* septicemia, transaminitis and hyperbilirubinaemia. MRCP was performed. (**a**) Axial *T*_2_-SSFSE showed “loss of signal void” in the posterior sectoral branch of the right portal vein (arrow). (**b**) *T*_1_ 3D-VIBE showed corresponding minimal T1-hyperintensity (arrow). (**c**) On DWI (*b* = 1000), marked hyperintensity was seen (arrow). (**d**) ADC map showed corresponding moderate hypointensity (arrow), indicating restricted diffusion. (**e**) Subsequent contrast-enhanced CT scan showed filling defect in the posterior sectoral branch of the right portal vein (arrow). This was of relatively low attenuation and did not show mass effect. In the context of septicemia, clinical diagnosis was that of portal pyemia. ADC, apparent diffusion coefficient; DWI, diffusion-weighted imaging; SSFSE, single-shot fast spin echo; VIBE, volume interpolated breath-hold examination.

Patient C was a 77-year-old lady who presented with acute onset fever and vomiting. Laboratory findings shows mild leucocytosis with markedly raised CRP of 219.8 mg L^−1^ (0–5). Liver function tests were deranged with transaminitis and a raised serum bilirubin of 138 umol L^−1^ (5–30). Blood cultures yielded *E. coli* bacteraemia. Clinical impression was that of hepatobiliary sepsis. MRCP was performed to evaluate for biliary dilatation. There was no biliary dilatation. However, there was suggestion PVT in the posterior sectoral branch of the right portal vein showing loss of signal void on *T*_2_WI, very mild T1-hyperintensity with marked restricted diffusion (Figure 4a–d). Contrast-enhanced CT scan was subsequently performed for further evaluation which confirmed filling defect in the posterior sectoral branch of the right portal vein (Figure 4e). Given the clinical presentation and *E. coli* bacteraemia, imaging diagnosis was that of portal pyemia. Patient was treated with intravenous antibiotics with clinical improvement as well as improvement in the liver function tests. The patient did not attend a scheduled follow-up CT scan.

## Case 4

**Figure 5.**
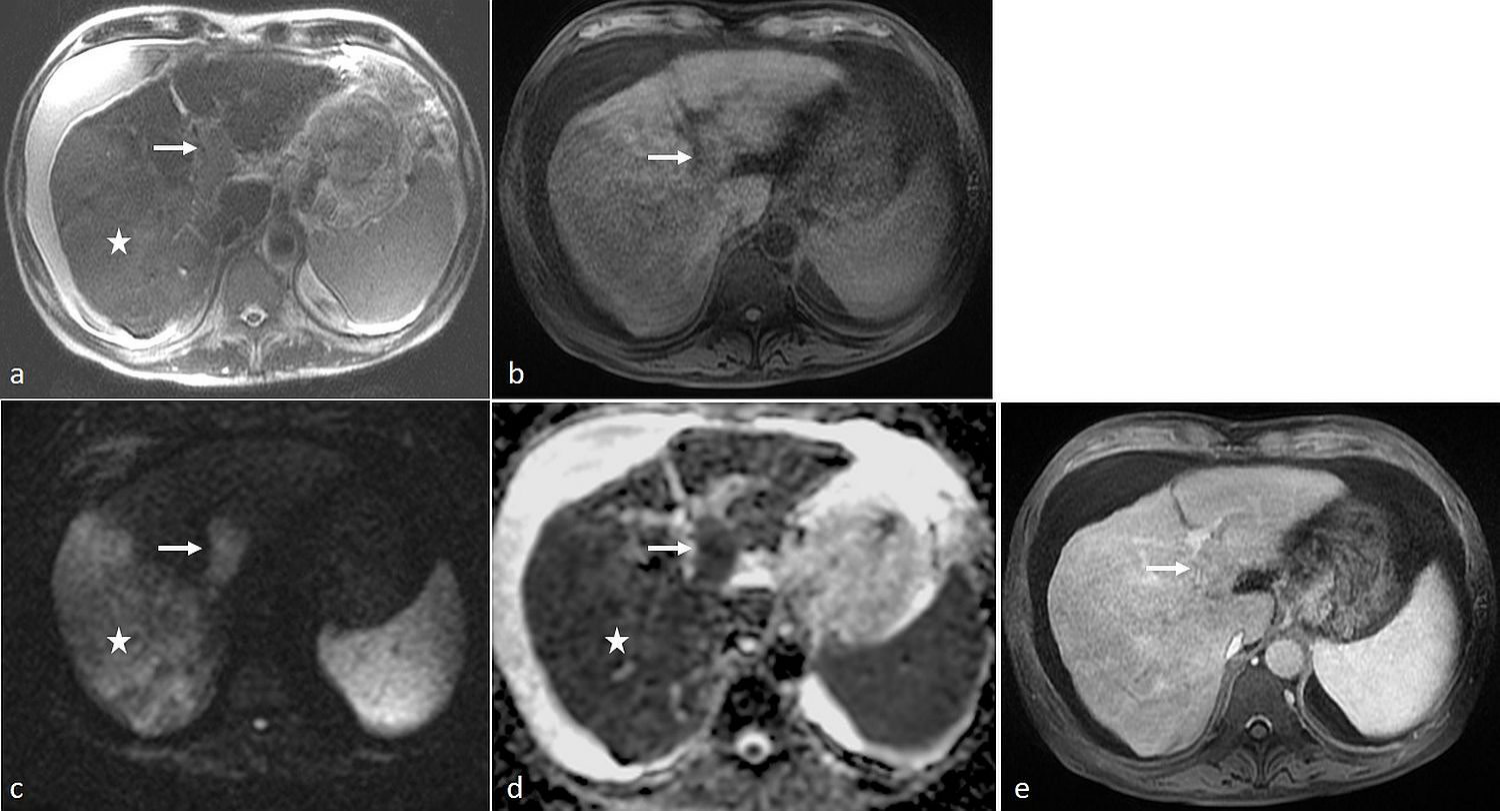
A 68-year-old gentleman presented with jaundice and abdominal distension. MRI was performed with intravenous administration of Dotarem. (**a**) On axial *T*_2_-SSFSE sequence, there was “loss of signal void” in both right and left portal veins, which demonstrated intermediate T2-hyperintensity with expanded appearance (arrow). There was also contiguous malignant-looking mass in the right lobe (star). (**b**) On *T*_1_ 3D-VIBE, there was corresponding mild heterogeneous T1-hyperintensity arising from the portal veins (arrow). (**c**) On DWI (*b* = 1000), marked hyperintensity was seen within the portal veins (arrow). The adjacent tumour mass in the right hepatic lobe also showed similar DWI hyperintensity (star). (**d**) ADC map showed corresponding marked hypointensity (arrow), similar to that of the right hepatic lobe tumour (star), indicating marked restricted diffusion. Findings on unenhanced MRI sequences were suspicious for tumour thrombus in the portal veins. (**e**) After intravenous contrast administration, presence of tumour thrombosis was confirmed, with the filling defect in the portal veins demonstrating heterogeneous enhancement (arrow). ADC, apparent diffusion coefficient; DWI, diffusion-weighted imaging; SSFSE, single-shot fast spin echo; VIBE, volume interpolated breath-hold examination.

Patient D was a 68-year-old gentleman with a history of cirrhosis secondary to chronic hepatitis B, who presented with jaundice and abdominal distension. Contrast-enhanced MRI was performed for further evaluation. On the unenhanced MRI sequences, a mass was seen in the right hepatic lobe, and within the portal veins there was loss of signal void with intermediate signal on *T*_2_WI, venous expansion and restricted diffusion (Figure 5a–d). This was compatible with tumour thrombus. Contrast-enhanced sequences after intravenous administration of Dotarem confirmed imaging diagnosis of hepatocellular carcinoma (HCC) in the right lobe with tumour thrombosis of the portal veins (Figure 5e). The patient was deemed not a candidate for surgery or chemotherapy and was placed on palliative care.

## Case 5

**Figure 6.**
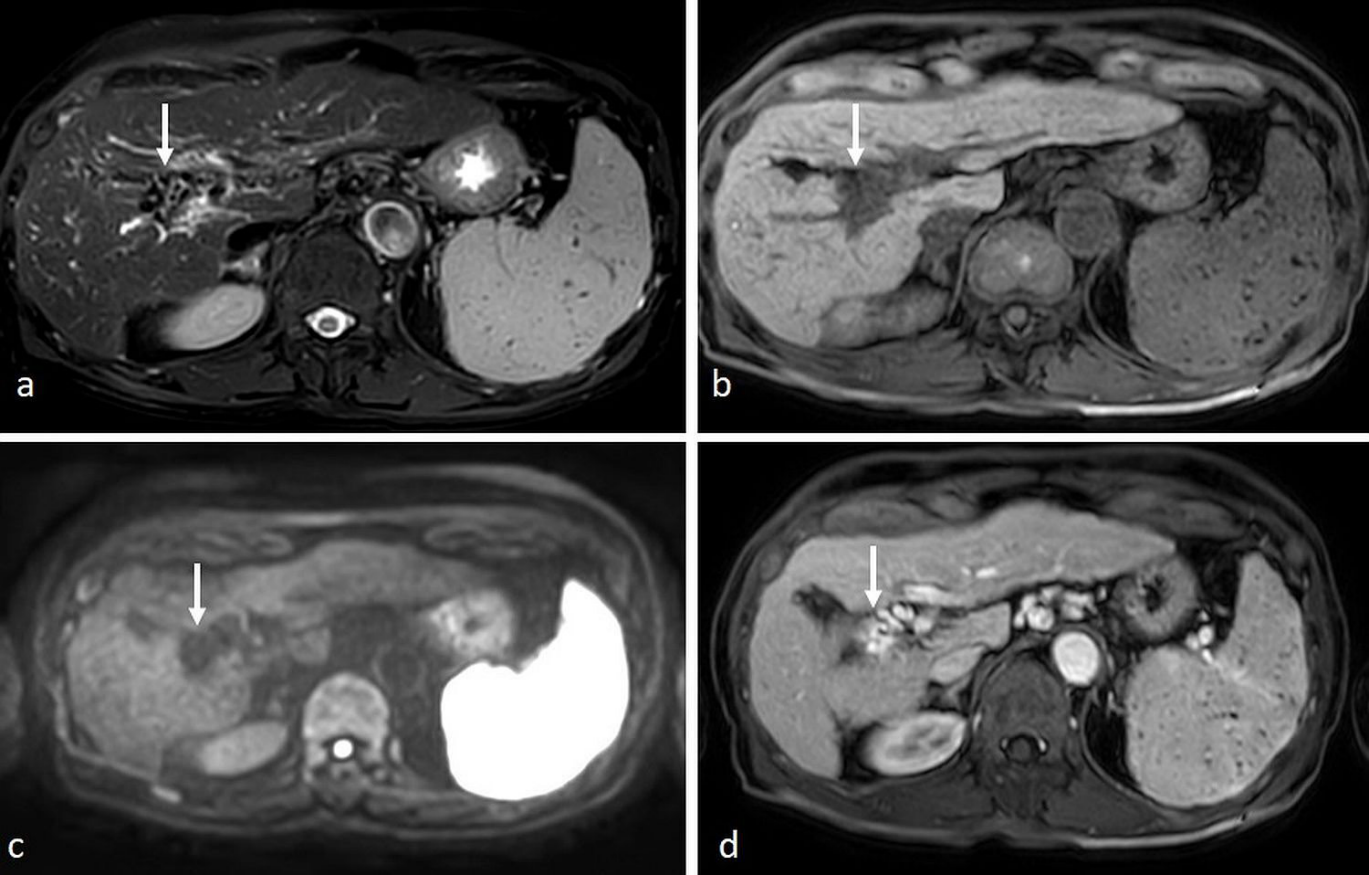
A 74-year-old gentleman with cirrhosis underwent MRI with Primovist for evaluation of a small hepatic lesion on surveillance ultrasound. (**a**) On axial *T*_2_-FS sequence, normal portal vein anatomy could not be seen. At the porta hepatis, there was a cluster of serpinginous signal voids (arrow) at the expected position of the right portal vein. (**b**) On *T*_1_ 3D-VIBE, there was corresponding hypointense appearance (arrow). (**c**) On DWI (b1000), there was no restricted diffusion and there was absent signal in this region (arrow). Findings on unenhanced MRI sequences, particularly *T*_2_WI, were suggestive of cavernous transformation of the portal vein. (**d**) After intravenous administration of contrast, mass-like enhancement of a tangle of vessels was seen at the expected position of the right portal vein (arrow), confirming cavernous transformation of the portal vein. There was also splenomegaly with multiple T2- and T1-hypointense foci in the spleen likely representing Gamma-Gandy bodies in keeping with portal hypertension, often seen in cavernous transformation of the portal vein. DWI, diffusion-weighted imaging; SSFSE, single-shot fast spin echo; VIBE, volume interpolated breath-hold examination.

Patient E was a 74-year-old gentleman with history of liver cirrhosis secondary to chronic hepatitis B. Surveillance hepatobiliary ultrasound showed a small right hepatic lobe hyperechoic nodule for which contrast-enhanced MRI was performed for further evaluation. No corresponding lesion was seen on MRI. However, on the unenhanced MRI sequences, there were features suggestive of cavernous transformation of the right portal vein (Figure 6a–c). Contrast-enhanced sequences after intravenous administration of Primovist confirmed imaging diagnosis of cavernous transformation of the right portal vein (Figure 6d).

## Case 6

**Figure 7.**
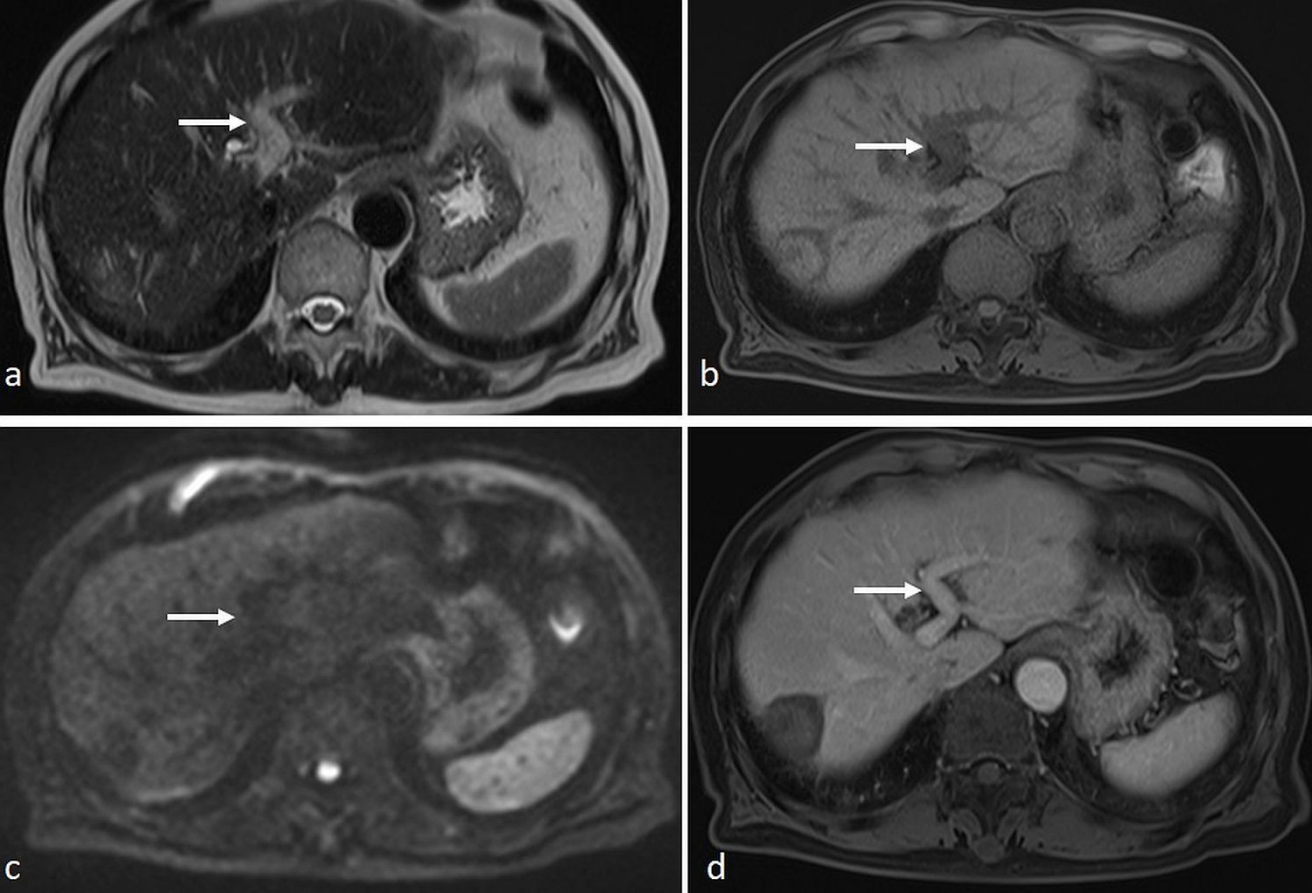
An 82-year-old gentleman with prior radiofrequency ablation of segment 7 HCC underwent MRI with Primovist. (**a**) On axial *T*_2_-SSFSE sequence the left portal vein appeared hyperintense (arrow) suggesting “loss of signal void”. (**b**) On *T*_1_ 3D-VIBE, normal hypointense appearance of the left portal vein was seen (arrow). (**c**) On DWI (*b* = 1000), there was no restricted diffusion (arrow). (**d**) There was normal enhancement of the left portal vein after intravenous contrast administration, without evidence of thrombosis. Apparent “loss of signal void” on *T*_2_-SSFSE was artefactual. DWI, diffusion-weighted imaging; HCC, hepatocellular carcinoma; SSFSE, single-shot fast spin echo; VIBE, volume interpolated breath-hold examination.

Patient F was an 82-year-old gentleman with a history of chronic hepatitis B. He had undergone prior radiofrequency ablation of a segment seven HCC. Contrast-enhanced MRI was performed with Primovist for routine surveillance. Axial *T*_2_-SSFSE showed loss of signal void in the left portal vein, but with normal appearance on *T*_1_ 3D-VIBE and DWI (Figure 7a–c). After intravenous contrast administration with Primovist, normal opacification of the left portal vein was seen (Figure 7d). Apparent loss of signal void on *T*_2_WI was artefactual.

## Case 7

**Figure 8.**
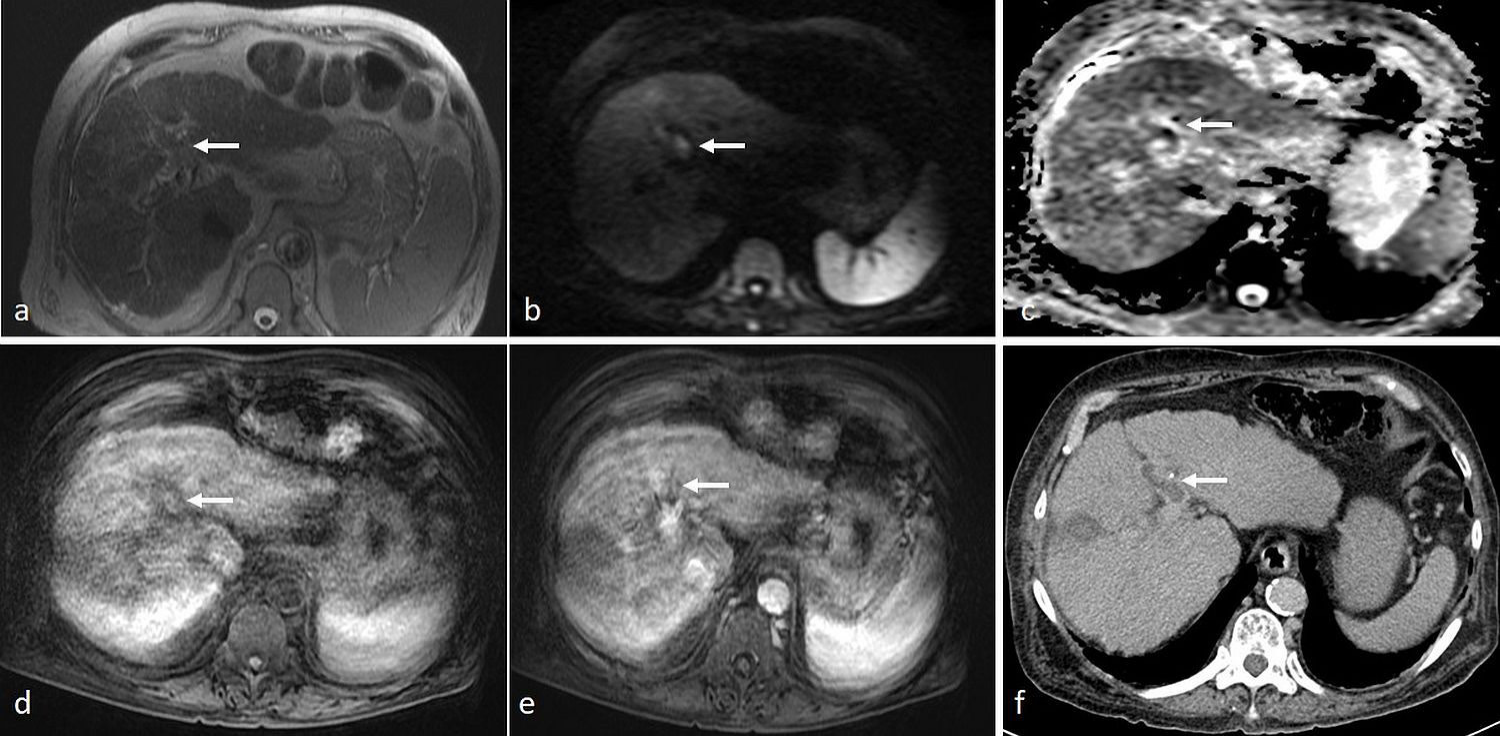
A 79-year-old gentleman underwent prior surgical resection and radiofrequency ablation procedures for recurrent HCC. MRI was performed with intravenous administration of Primovist. (**a**) On axial *T*_2_-SSFSE there was loss of signal void in the left portal vein, which appeared of intermediate signal (arrow). (**b**) On DWI (*b* = 1000), there was corresponding hyperintensity on DWI (arrow). (**c**) On ADC map, there was corresponding hyperintensity suggestive of T2 shine-through (arrow). (**d**) On *T*_1_ 3D-VIBE, mild T1-hyperintensity was seen along the course of the left portal vein (arrow). (**e**) After intravenous contrast administration, a faint filling defect was seen in the left portal vein (arrow), although accurate assessment was limited by presence of breathing artefact. (**f**) Subsequent contrast-enhanced CT scan confirmed partial left portal vein thrombosis that did not show significant mass effect and was of low attenuation (arrow). Given presence of T2 shine-through on ADC map, lack mass effect and low attenuation on CT, findings were suggestive of acute bland PVT rather than tumour thrombus. ADC, apparent diffusion coefficient; DWI, diffusion-weighted imaging; HCC, hepatocellular carcinoma; PVT, portal venous thrombosis; SSFSE, single-shot fast spin echo; VIBE, volume interpolated breath-hold examination.

Patient G was a 79-year-old gentleman with alcoholic liver cirrhosis. He had undergone prior surgical resection and radiofrequency ablation procedures for recurrent HCC. Contrast-enhanced MRI was performed with intravenous administration of Primovist as part of routine surveillance. No tumour recurrence was seen. However, there was left portal vein thrombosis (Figure 8a–e). Subsequent contrast-enhanced CT performed 2 days later again showed partial thrombosis of the left portal vein (Figure 8f). Given the presence of DWI hyperintensity, there was uncertainty if this represented an acute bland thrombosis or tumour thrombus, as hyperintensity on DWI can either represent “T2 shine-through” or true restricted diffusion. Careful evaluation of the apparent diffusion coefficient (ADC) map on MRI showed corresponding hyperintensity suggestive of “T2 shine-through” effect rather than restricted diffusion (Figure 8c). Furthermore, based on appearance on subsequent contrast-enhanced CT and the absence of tumour recurrence, imaging diagnosis was acute bland PVT rather than tumour thrombus. Patient was managed conservatively, and subsequent contrast-enhanced CT scan showed stable PVT.

## Discussion

Clinical diagnosis of PVT is challenging due to non-specific symptoms such as abdominal pain, fever, nausea.^8,9^ As such, the diagnosis is often made incidentally on imaging. PVT is important to recognise as it has implications on patient management and prognosis. Risk factors for PVT include chronic liver disease (in particular liver cirrhosis), malignancy (in particular HCC, cholangiocarcinoma and pancreatic cancer), locoregional inflammatory conditions such as pancreatitis and cholecystitis, severe sepsis or pro-thrombotic states.^10^ With increasing use of thermal ablative techniques for minimally invasive treatment of liver malignancies, PVT is also recognised as a potential complication which usually manifests within a few days of the procedure.^11^

In patients with severe chronic renal impairment where intravenous contrast is contraindicated, diagnosis of PVT can be made on NC-MRI or Doppler ultrasound. However, ultrasound has inherent limitations. In particular, image quality can be variable due to operator-dependence and patient factors such as habitus and ability to comply with scanning. Therefore, radiologists should be cognizant of the appearance of PVT on NC-MRI, in patients whom intravenous contrast is contraindicated.

In particular, radiologists should always evaluate the portal veins for incidental PVT, on standard NC-MRI sequences of *T*_2_WI, *T*_1_WI and DWI. On *T*_2_WI, it is important to evaluate for normal signal void of flowing blood. Loss of signal void, usually seen as T2-hyperintensity, raises suspicion for PVT. Furthermore, *T*_2_WI is useful for differentiating tumour thrombus from acute bland PVT. Intermediate T2-signal intensity and mass effect with venous expansion is typically seen with tumour thrombus, while acute bland PVT is usually more T2-hyperintense and the branching configuration and signal intensity may even simulate dilated intrahepatic bile ducts.^3^

On *T*_1_WI, T1-hyperintensity is suggestive of acute PVT due to the presence of methaemoglobin leading to shortening of T1-relaxation time.^12^ *T*_1_WI has also been shown to be able to differentiate between acute and chronic PVT as the T1-hyperintensity in acute PVT reduces in signal over time.^13^ On *T*_1_WI, tumour thrombus is usually hypointense similar to tumour signal, in contrast to the T1-hyperintensity in acute bland PVT.

DWI is an established functional imaging technique in abdominal imaging and is a useful sequence for detection of PVT. With advances in MRI techniques, DWI improves detection of pathology and lesion characterisation, particularly where intravenous contrast is contraindicated or post-contrast images degraded by artefact.^14^ Appearance of acute PVT on DWI is variable. Acute thrombus itself usually does not demonstrate restricted diffusion, however thrombus containing higher trapped red blood cell content, may demonstrate restricted diffusion, particularly within the first week, with progressive reduction in signal beyond that.^15^ DWI is particularly useful in differentiating tumour thrombus from bland thrombus, with tumour thrombus shown to demonstrate greater restricted diffusion with lower ADC values, compared to acute bland PVT.^16^ Further, DWI hyperintensity in acute bland PVT can also be attributed to T2 shine-through due to presence of T2-hyperintensity of the acute thrombus.^17^ Careful visual evaluation of the ADC map will demonstrate corresponding hyperintensity suggesting “T2 shine-through”. This can also help differentiate from tumour thrombus, which shows true restricted diffusion (corresponding hypointensity on ADC map). In contrast, portal pyemia and tumour thrombus both can show marked restricted diffusion, therefore evaluation for other imaging features of malignancy such as contiguous tumour masses in the liver parenchyma, venous expansion and clinical presentation can help differentiate both entities.

Both Patient A and Patient B presented with bland PVT, with loss of signal void on *T*_2_WI. However, in Patient B restricted diffusion was seen, while in Patient A there was no restricted diffusion. This is due to different ages of the PVT, which is likely to be more acute in Patient B.

In portal pyemia, pus develops within portal venous system. This is usually as a result of severe intra-abdominal sepsis, most commonly secondary to diverticulitis, with resultant bacterial seeding into the portal venous system.^18^ When associated with bacteraemia, presence of thrombosis and gas within portal venous system on contrast-enhanced CT is diagnostic, although cases of portal pyemia without presence of portovenous gas have also been reported.^19,20^ While there have been no reported MRI features specific for portal pyemia, DWI can show restricted diffusion due to presence of pus, and in the appropriate clinical setting, this can aid in differentiating from bland PVT. However, as acute bland PVT can also show restricted diffusion within the first week, this finding should always be correlated the patient’s clinical presentation.

Tumour thrombosis of the portal vein is seen as intermediate signal within the venous system on *T*_2_WI, with expansion of the affected vessels as well as restricted diffusion on DWI. Tumour thrombosis of the portal vein is typically associated with HCC. Tumour thrombosis in HCC is associated with more aggressive tumour grade and classifies the patient at an advanced stage where mainstay of treatment is palliative.^21^ Gadoxetic contrast-enhanced MRI has been shown to be superior to contrast-enhanced CT for detection of tumour thrombosis of the portal vein in HCC, and it has been suggested that the added value of *T*_2_WI and DWI contributes to diagnostic performance.^22^ Therefore, recognition of tumour thrombus, even on NC-MRI utilising *T*_2_WI and DWI, is important for patient management.

Cavernous transformation of the portal vein most commonly occurs in chronic PVT when collaterals develop around the thrombosed portal vein, usually around the porta hepatis. It may also be secondary to rarely aetiologies such as obliterative portal venopathy or congenital absence of the portal vein.^23^ On MRI, a mass-like tangle of vessels with beaded appearance representing collateral hepatopetal venous channels is seen at the expected location of the portal vein, best depicted on the contrast-enhanced sequences.^23^ On *T*_2_WI, the patent collateral venous channels can be seen as serpinginous signal voids. The portal vein thrombus itself may not be directly visualised on MRI but, despite venous collaterals, there is still often evidence of portal hypertension such as ascites, portosystemic varices and splenomegaly.^24^

Radiologists should also be aware of pitfalls when evaluating NC-MRI for PVT. Firstly, on *T*_2_WI, the normal signal void of patent portal veins may not always be present. Loss of signal void can be seen in sluggish flow in an otherwise patent vessel, in-plane flow, or entry-slice phenomenon where flowing blood entering an image slice appears hyperintense due to unsaturated spins.^25,26^ Therefore, other planes and sequences should be carefully assessed. Secondly, on DWI, acute bland PVT, tumour thrombus and portal pyemia can all show DWI hyperintensity and make differentiation on NC-MRI challenging. In fact, some studies have suggested that DWI in such cases may not be useful in differentiating acute bland PVT from tumour thrombus.^27,28^ However, careful evaluation of ADC map may demonstrate “T2 shine-through” that can be seen in acute bland PVT, and this finding can differentiate acute bland PVT from tumour thrombus or portal pyemia.

In conclusion, recognition of incidental PVT on unenhanced MRI is important for patient management. In practice, DWI can sometimes be the first clue to the presence of PVT and prompt closer evaluation of other anatomical sequences. However, as there can be overlap of NC-MRI imaging features between bland thrombus, portal pyemia and tumour thrombus, correlation with the appropriate clinical context is still necessary.

## Learning points

1. Acute PVT is seen on NC-MRI as loss of signal void *T*_2_WI and hyperintensity on *T*_1_WI, and can sometimes show restricted diffusion on DWI.
2. DWI can help differentiate bland thrombus from portal pyemia in the appropriate clinical context. Hyperintensity on both DWI and ADC map indicates T2 shine-through that would more likely suggest acute bland PVT rather than tumour thrombus or portal pyemia. However, clinical correlation is still necessary.
3. Tumour thrombosis of the portal veins shows intermediate signal on *T*_2_WI and causes expansion of the portal veins. These features can help differentiate between tumour thrombus from bland thrombus or portal pyemia.
4. On MRI, cavernous transformation of the portal vein is seen as a tangle of vessels at the porta hepatis that can appear mass-like.
5. Loss of flow void on *T*_2_WI can be artefactual, and other sequences should be evaluated carefully.
